# Changes of Exhaled Volatile Organic Compounds in Postoperative Patients Undergoing Analgesic Treatment: A Prospective Observational Study

**DOI:** 10.3390/metabo10080321

**Published:** 2020-08-07

**Authors:** Benjamin Löser, Alina Grabenschröer, Giovanni Pugliese, Pritam Sukul, Phillip Trefz, Jochen K Schubert, Wolfram Miekisch

**Affiliations:** 1 Department of Anesthesiology, Center of Anesthesiology and Intensive Care Medicine, University Medicine Rostock, Schillingallee 35, 18057 Rostock, Germany; alina.grabenschroeer@uni-rostock.de (A.G.); jochen.schubert@uni-rostock.de (J.K.S.); 2 Rostock Medical Breath Research Analytics and Technologies (ROMBAT), Department of Anesthesiology and Intensive Care, Rostock University Medical Center, Schillingallee 35, 18057 Rostock, Germany; giovanni.pugliese@uni-rostock.de (G.P.); pritam.sukul@uni-rostock.de (P.S.); phillip.trefz@uni-rostock.de (P.T.); wolfram.miekisch@uni-rostock.de (W.M.)

**Keywords:** anesthesia, VOC, pain, painkiller, breath analysis, breath biomarkers, PTR-MS

## Abstract

Assessment and treatment of postoperative pain can be challenging as objective examination techniques to detect and quantify pain are lacking. We aimed to investigate changes of exhaled volatile organic compounds (VOCs) in patients with postoperative pain before and after treatment with opioid analgesics. In an observational study in 20 postoperative patients, we monitored for postoperative pain, hemodynamic parameters, and catecholamines before and during treatment. VOCs in the patients were determined by direct real-time proton transfer reaction time-of-flight mass spectrometry prior (0 min) and after piritramide application (15 min as well as 30 min). Cardiovascular variables changed and norepinephrine levels decreased during treatment. The VOCs acetonitrile (<0.001), acetaldehyde (*p* = 0.002), benzopyran (*p* = 0.004), benzene (*p* < 0.001), hexenal (*p* = < 0.001), 1-butanethiol (*p* = 0.004), methanethiol (*p* < 0.001), ethanol (*p* = 0.003), and propanol (*p* = < 0.001) changed significantly over time. Patients with Numeric Rating Scale (NRS) < 4 showed a significantly lower concentration of hexenal compared to patients with NRS > 4 at the time points 15 min (45.0 vs. 385.3 ncps, *p* = 0.047) and 30 min (38.3 vs. 334.6 ncps, *p* = 0.039). Breath analysis can provide additional information for noninvasive monitoring for analgesic treatment in postoperative patients.

## 1. Introduction

Pain is a complex sensation with multiple definitions: The International Association for the Study of Pain (IASP) defines it as “an unpleasant sensory and emotional experience associated with actual or potential tissue damage, or described in terms of such damage”, whereas McCaffery describes pain as “whatever the person experiencing it says it is, and exists whenever the person experiencing it says it does” [1,2,3]. It is estimated that more than 230 million surgeries are performed worldwide every year [4]. Although improvements in pain treatment could be achieved in the past, patients frequently suffer from postoperative pain [5,6].

Due to the subjective nature of pain, assessment and treatment can be challenging for physicians. For the assessment of pain, intensity scores such as the Verbal Rating Scale (VRS), Visual Analogue Scale (VAS), and Numerical Rating Scale (NRS) are used regularly [7]. Nevertheless, these unidimensional and subjective means reflect rather the subjectivity of pain and the patients’ individual sensation than being an objective tool for the practitioner. Measurement methods based on objective (biochemical) and/or metabolic parameters to detect pain are not available yet. Care, especially in vulnerable patient groups such as ventilated and sedated or physically and/or mentally disabled patients, might be improved by noninvasive point of care testing for pain.

Analysis of volatile organic compounds (VOCs) is a novel noninvasive method [8] which can detect hundreds of VOCs with relation to physiological and pathophysiological conditions in the human body [9,10,11,12]. Changes in VOCs concentrations during exhalation may indicate changes in the body’s chemistry. These changes can be associated with metabolic disorders [13] or may be used for therapeutic drug monitoring [14]. Furthermore, volatile biomarkers indicating oxidative and metabolic stress in patients can be detected [15,16].

Objective and noninvasive examination techniques to detect and quantify pain are lacking. We therefore aimed to investigate the changes of exhaled VOCs in patients with postoperative pain before and after treatment with opioid analgesics.

## 2. Results

### 2.1. Patients’ Characteristics and Outcome

We included 20 patients (convenience sampling). The patients’ characteristics are shown in Table 1.

Table S1 demonstrates the individual pre-existing conditions and the specific indications for the performed surgeries for each patient.

#### 2.1.1. Changes in Exhaled VOCs

Table 2 shows VOCs which were preselected based on a noticeably higher expiratory concentration compared to inspiratory values. Baseline values (0 min) and changes of VOCs (15 min and 30 min) as well as the corresponding statistical significances are illustrated.

**Figure 1:**
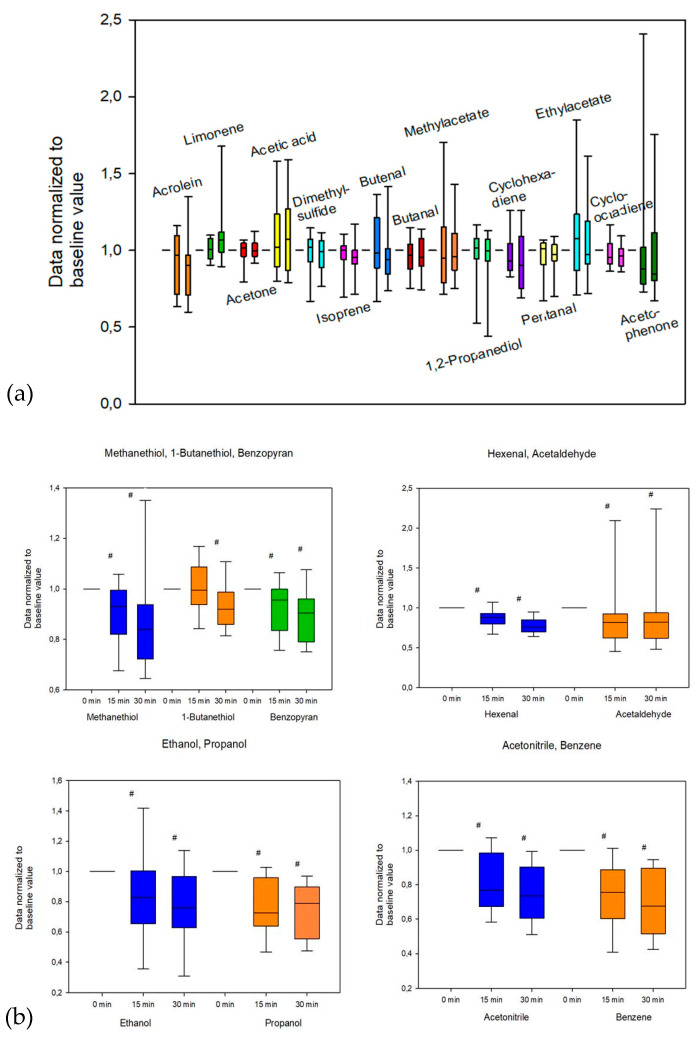
Normalized changes of VOCs. (**a**) The figure illustrates the distribution of data during the measurement time points (0 min, 15 min, and 30 min). Data were normalized to baseline values to show relative changes. (**b**) The figure illustrates the VOCs methanethiol, 1-butanethiol, benzopyran, ethanol, propanol, acetonitrile, benzene, hexenal, and acetylaldehyde, which were found to have significant changes during the observation period. Data were normalized to baseline values to show relative changes; hashes (#) indicate statistically significant changes.

Relative changes (%) of the examined VOCs are shown in Table S2. In short, acetonitrile, acetaldehyde, benzopyran, benzene, hexenal, 1-butanethiol, methanethiol, ethanol, and propanol showed significant changes over time (Table 2). Boxplots of the corresponding VOCs are shown in Figure 1.

#### 2.1.2. Changes in Cardiovascular Dynamics

Detailed statistical analysis of cardiovascular dynamics between baseline (0 min) and after 15 and 30 min showed statistically significant differences regarding the median of Cardiac Index (CI), Systolic Arterial Pressure (SAP), and Mean Arterial Pressure (MAP) (Table S3). Heart Rate (HR), Diastolic Arterial Pressure (DAP), and Stroke Volume Index (SVI) did not change in a statistically significant way. The individual course of the cardiovascular variables of the observed patients is illustrated in Figure S1. Four patients had to be excluded from hemodynamic monitoring due to technical monitoring problems.

#### 2.1.3. Catecholamine Levels in Plasma

Results of patients’ catecholamine levels in plasma are presented in Table 3. Blood samples could be taken from 18 of the 20 observed patients. In two cases, it was not possible to aspirate blood from intravascular catheters.

No statistically significant differences regarding dopamine and epinephrine could be found, whereas a significant difference in patients’ norepinephrine levels could be observed (Table 3).

#### 2.1.4. Correlation Analysis

The performed correlation analyses for the variables “catecholamine levels”, “cardiovascular variables”, and “VOC levels” did not show close correlations (Table S4).

#### 2.1.5. Comparison of Patient Groups Using the NRS

We divided patients into two groups: patients with NRS ≤ 4 and patients with NRS > 4 were specifically examined in a subgroup analysis regarding changes of VOCs: patients with a recorded NRS ≤ 4 (time point 15 min) showed a significantly lower concentration of hexenal compared to patients with NRS > 4 at the time points 15 min (45.0 vs. 385.3 ncps, *p* = 0.047) and 30 min (38.3 vs. 334.6 ncps, *p* = 0.039).

## 3. Discussion

With this prospective observational study, we examined whether changes of VOCs might be helpful in determining and quantifying pain in postoperative patients undergoing analgesic treatment. We therefore measured and compared VOCs at different time points (0 min, 15 min, and 30 min) and found significant changes in the alveolar concentrations of several substances and catecholamine levels in plasma.

High levels of ethanol and propanol could be found via breath analysis in the observed patients. Both substances declined after application of analgesics. Nevertheless, propanol and ethanol are most probably “exogenously administered” substances in the observed patients and are elements of disinfectant solutions, which were absorbed by the patients prior to surgery and are subject to a wash out phase in the Post Anesthesia Care Unit (PACU) later on. Nevertheless, ethanol may also be produced by gut bacteria, transported via the blood stream into the lung and exhaled in human breath. As its release may also be related to bronchial circulation [17], it is possible that bronchial vasoconstriction (norepinephrine rise at 30 min) was also related to a decreased ethanol output.

Benzopyran is a heterocyclic organic compound. It may induce genotoxicity [18]. Other studies indicate that benzopyran derivatives may be useful anti-inflammatory agents [19]. Additionally, an antinociceptive effect for 6-isobutyryl-5,7-dimethoxy-2,2-dimethyl-benzopyran was reported in mice mediated by an opioid-like mechanism [20]. The origin of the detected benzopyran in breath is unknown. It is likely that benzopyran is a metabolite of prior administered drugs. This would be in line with the observed wash out kinetic.

In our study, 6 of 20 patients were known to be smokers. Smoking is associated with increased breath levels of acetonitrile and benzene [21]. Usually benzene levels in smokers rapidly decrease (within one hour) to non-smokers levels, whereas acetonitrile levels remain elevated in smokers for several days [21,22]. Surprisingly, even after ceasing smoking for several hours a further decrease of benzene could be detected during breath analysis in this study. This observation demonstrates the high sensitivity of proton transfer reaction time-of-flight mass spectrometry (PTR-MS). As these two VOCs are associated with nicotine abuse we believe that this finding itself is not associated with treatment and the application of piritramide.

Methanethiol, being a volatile sulfur-containing compound, can be found in various mammalian matrices (e.g., blood, breath, feces, and tissues) and is known for its toxicity and bad odor [23]. Dimethyl-sulfide as well as 1-butanethiol appear to be major components of bacterial metabolism.

C_2_H_6_S and C_4_H_10_S are potentially originated from gut bacterial methylation [23]. Gut bacteria regulate the activity of ß-glucuronidase and ß-lyase enzymes [24], which reversibly control the methylation process. Administrations of non-steroidal anti-inflammatory drugs [25], morphine [26], steroids, and contraceptives [27] downregulate the natural activity of these enzymes and may cause dysbiosis of gut microbiota [28]. The decrease of volatile sulfur compounds may therefore be related to the downregulation of the methylation process by the administered analgesic therapy.

Catecholamine levels in plasma were analyzed using High-Performance Liquid Chromatography (HPLC). Epinephrine and dopamine did not show significant changes during our observation period. Nevertheless, norepinephrine showed significant changes over time. Although the median for norepinephrine at 30 min is higher than the median at 0 min, a comparison of the corresponding means showed a decline of norepinephrine at 30 min (3.7 nmol/L) compared to the time point 0 min (13.0 nmol/L). This observation would be in line with the recorded hemodynamic parameters which also showed a significant decrease in arterial blood pressures showing a correlation between analgesic treatment, norepinephrine levels in plasma, and a lower arterial blood pressure in the observed patients. 

Significant reductions in the concentration of two aldehydes (hexenal and acetaldehyde) in breath could be found. Aldehydes, which can be found in blood, secretions, and breath [11], are of special interest in VOC analysis due to their relationship to metabolic functions. It is known that ethanol metabolism can lead to increased levels of acetaldehyde [29,30,31]. Therefore, it might be assumed that high ethanol levels could be partly responsible for higher concentration levels of acetaldehyde which, similar to propanol and ethanol, also decreased over time. Promyelocytic cells and isolated neutrophils emit acetaldehyde [32,33]. Acetaldehyde might be responsible for the relaxation of isolated blood vessels by modulating calcium channels [34] but may also cause vasoconstriction by releasing endogenous catecholamines [35].

Nociception and the activation of the sympathetic nervous system involve transmitters such as epinephrine, norepinephrine, or glucocorticoids, which increase heart rate, stroke volume, and peripheral resistance [36]. As we found a significant decrease in acetaldehyde in breath, this observation might be explained by a lower sympathetic activation after analgesic treatment as a necessarily reduced emission of acetaldehyde might have led to a starting relaxation of blood vessels. This assumption would be supported by the clinically not relevant but significantly lower arterial pressures, which were recorded in our patient cohort.

Aldehydes are well-known markers for oxidative stress in humans and plants [37,38,39,40]. As with other aldehydes, hexenal is a breakdown product of fatty acids [41]. At low pH values it is hydrolyzed to hydroxyl-hexanal. Hexenal showed a significant reduction over time in patients who underwent analgesic treatment with piritramide. This observation might be explained by lower stress levels and less resulting oxidative stress in patients with reduced pain levels. This assumption is further supported by a performed subgroup analysis which also showed that patients with “more pain” (NRS > 4) had higher hexenal levels compared to patients with “less pain” (NRS ≤ 4). Therefore, hexenal and acetaldehyde could be directly associated with successful analgesic treatment as metabolic and stress levels are reduced due to a resulting lower pain level by the application of piritramide in the observed patients. Possibly, these two VOCs might be a valuable progression parameter for the successful treatment of patients’ pain.

This study has limitations. First of all, because of the small sample size one should refrain from drawing definite conclusions regarding the analgesic treatment of postoperative patients and associated specific (pattern) changes of VOCs based on our study. Furthermore, the monocentric study design limits the generalizability of our findings. As there are many analytical problems associated with plasma catecholamine determination [42], variation and deviation from true values of serum catecholamine levels might limit the significance of these results. Some of the VOCs observed in the study may be related to similar metabolic pathways in the human body. Therefore, the time courses of some VOCs during treatment may not be completely independent from the course of other substances. It is known that age and gender may affect the concentrations of some VOCs. However, as we did not compare absolute VOC concentrations between the patients—in our study each patient served as his or her own control—those effects are considered as negligible. We therefore monitored concentration changes for each patient, mirroring acute effects of treatment rather than (chronic) effects of the patient’s diseases. 

Although patients showed broad variations in clinical parameters, we observed interesting concentration changes in a number of volatile metabolites. Acetaldehyde and hexenal significantly decreased after analgesic treatment indicating that oxidative and metabolic stress decrease in patients after treatment with piritramide. Breath analysis might therefore add helpful information as progression parameter for analgesic treatment in postoperative patients as objective methods to measure and quantify pain are still lacking [43].

## 4. Materials and Methods

The Institutional Ethics Committee of the Medical Board of the University of Rostock, Germany approved this study (A 2018-0097) on the 30th of May 2018. All patients gave written informed consent.

### 4.1. Design of the Study and Patients

This single-center observational study was carried out in March 2019 at the Department of Anesthesiology at the University Medicine Rostock. We included 20 postoperative patients (32–76 years) with American Society of Anesthesiologists (ASA) physical status class I–III.

Postoperatively patients were transferred to the PACU and received standard postoperative care. Patients were frequently monitored for postoperative pain using the NRS. VOCs were measured using direct real time proton transfer reaction-time-of-flight mass spectrometry (PTR-ToF-MS) analysis as previously described [44]. In short, patients were positioned in their beds in a 30 degrees elevated resting position and were breathing normally and relaxedly with respiratory rates of 10–18 through a sterile t-piece without major restriction. PTR-ToF-MS was set to a time resolution of 200 ms and a sampling flow of 20 mL/min. The drift voltage was 610 V, the drift temperature was 75 °C, and the drift tube pressure was 2.3 mbar, resulting in an E/N ratio of 138 Td. Mass scale was recalibrated after every run of 60 s. Masses used for that purpose were 21.023 (H3O+-Isotope), 29.998 (NO+), and 59.049 (protonated acetone). Data were processed via PTR-MS viewer version. 3.2.8 (IONICON Analytik GmbH, Innsbruck, Austria) and expiratory and inspiratory phases were recognized by means of a custom-made data processing algorithm named “breath tracker” (MATLAB version 7.12.0.635, R2011a) [44]. 

We did not apply separate sampling procedures (in bags or traps) before analysis, as this kind of sampling may cause several biases in the results [45,46], but used a direct online approach; the patient is breathing normally and relaxedly through a mouthpiece without major restriction. The breath sample is continuously drawn into the PTR-TOF through a t-piece and a (heated) transfer line. The low restriction and the limited flow of the sidestream sampling line (20 mL/min) do not affect breathing patterns or VOC profiles [44]. Therefore, potential biases caused by unphysiological breathing maneuvers [47,48], restrictions in the sampling system [49], or storage effects can be avoided. This kind of direct sampling technique was investigated in any depth [50,51,52], has been proven to be reliable, and has successfully been applied in several (clinical) studies [13,27,53]. As the patients of this study were breathing normally at respiratory rates between 10 and 18 breaths per minute, ventilation rates did not vary significantly during the measurements.

Only VOCs with a signal-to-noise ratio of at least 3 (noise was determined via blank measurements) and a higher abundance in expiration compared to inspiration and differences between groups or time points were considered as potential marker substances. Expiratory abundance had to be above inspiratory abundance plus the standard deviation of inspiratory abundance. Only VOCs with changes over the observed time were selected for detailed analysis.

We furthermore continuously recorded hemodynamic variables (HR, CI, SAP, DAP, MAP, and SVI) using a noninvasive finger cuff-derived pulse wave analysis (ClearSight EV 1000 system, Edwards Lifesciences Corp.; Irvine, CA, USA). In addition, blood samples were taken to determine catecholamine levels in plasma.

Patients’ characteristics and clinical data such as medical preconditions were obtained from our digital data management program COPRA (COPRA System, Berlin, Germany) and electronic health records.

### 4.2. General Anesthesia

All operative procedures were carried out under general anesthesia with endotracheal intubation. Propofol and sufentanil or remifentanil were used for anesthesia induction. For anesthesia maintenance propofol or sevoflurane was used. Sufentanil or remifentanil were used intraoperatively as analgesics. Mallinckrodt Cuffed Lo-ContourTM tracheal tubes (Covidien, Dublin, Ireland) were used during anesthesia. Ventilation of the patients was performed using the anesthesia machine Primus (Dräger, Lübeck, Germany).

### 4.3. Postoperative Care

After surgery patients were transferred to the PACU. Patients received standard postoperative and analgesic care. In the case of NRS > 3, patients were treated with the opioid analgesic piritramide in a standardized way according to body weight (0.1 mg/kg with a maximum of 7.5 mg in patients < 70 years and a maximum of 3.75 mg in patients ≥ 70 years).

### 4.4. Study Measurements of VOCs, Cardiovascular Variables, and Catecholamine Concentration in Plasma

**Figure 2:**
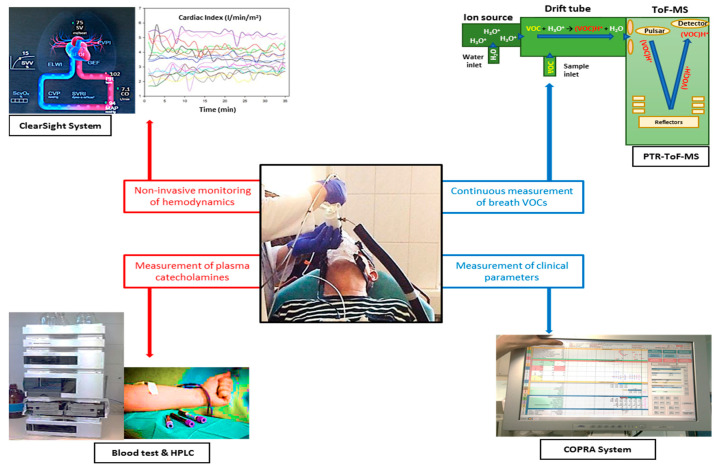
This figure summarizes the used equipment during the study. Patients’ characteristics and clinical data as well as hemodynamic parameters were obtained from our digital data management program COPRA. VOCs were measured using direct real-time proton transfer reaction time-of-flight mass spectrometry (PTR-ToF-MS) analysis. Blood samples were taken to determine catecholamine levels in plasma prior and after analgesic treatment and were subsequently analyzed using High-Performance Liquid Chromatography (HPLC). Hemodynamic variables were measured using noninvasive finger cuff-derived pulse wave analysis (ClearSight EV 1000).

We measured VOCs prior, 15 min, and 30 min after piritramide application. Hemodynamic variables were continuously recorded using noninvasive finger cuff-derived pulse wave analysis using the ClearSight EV 1000 system. Before and during the period of analgesic treatment the above-mentioned hemodynamic variables were recorded. Furthermore, blood samples for the determination of catecholamine (epinephrine, norepinephrine and dopamine) levels in plasma were taken prior and 30 min after analgesic treatment. Blood samples were anticoagulated with ethylenediaminetetraacetate (EDTA), immediately cooled down to 4 °C, and subsequently analyzed using HPLC [42]. Figure 2 illustrates the study set-up.

### 4.5. Statistical Analysis

Data were analyzed using SPSS 22.0.0 (SPSS Inc., Chicago, IL, USA) and SigmaPlot 13.0 (Systat Software Inc., San José, CA, USA). Descriptive results are given as median (25th; 75th percentile). Categorical data are presented as absolute and relative frequencies. Boxplots show VOCs results. The medians are presented as bold horizontal lines across the boxes. The lower and upper edges of the boxes show the 25th and 75th percentiles. Whiskers indicate the lowest and highest values within a 1.5 box length range away from the percentiles (25th and 75th).

We furthermore performed a statistical analysis to evaluate if pain intensity and analgesic treatment with piritramide correlated with changes of VOCs, cardiovascular variables, and catecholamine levels in plasma. For statistical differences between VOC concentrations and cardiovascular variables we used Friedman repeated measures analysis of variance (ANOVA) on ranks with consecutive Student–Newman–Keuls-corrected post hoc test for pairwise multiple comparison. Wilcoxon–Mann–Whitney test was used to compare two samples. Correlation analyses were performed using Pearson’s correlation test. A *p*-value of < 0.05 was considered to be statistically significant.

## 5. Conclusions

In this prospective observational study, we observed interesting VOC concentration changes in patients undergoing postoperative analgesic treatment. Acetaldehyde and hexenal significantly decreased after analgesic treatment indicating that oxidative and metabolic stress decrease in patients after treatment with piritramide. Although the study group is too small to generalize the findings on a clinical level, breath analysis might add important information to monitor analgesic treatment in postoperative patients.

## Figures and Tables

**Table 1:** Patients’ characteristics.

**Demographics**	
sex (male/female)	15/5
age (year)	60 (48;67)
height (cm)	175 (172;182)
body weight (kg)	88 (67.2;104.3)
body mass index	29.5 (21.8;33.3)
body surface (m^2^)	2.05 (1.8;2.26)
**ASA Physical Status Classification (Grade)**	
I	15% (3/20)
II	35% (7/20)
III	50% (10/20)
**Permanent Medication**	
non-opioid analgesics	40% (8/20)
opioids	20% (4/20)
blood thinners	35% (7/20)
statins	25% (5/20)
antihypertensives	45% (9/20)
diuretics	35% (7/20)
thyroid hormones	15% (3/20)
**Pre-existing Medical Conditions**	
lung diseases	15% (3/20)
cardiovascular diseases	70% (14/20)
renal failure	15% (3/20)
diabetes mellitus	5% (1/20)
history of smoking	30% (6/20)
**Type of Surgery**	
general surgery/visceral surgery	45% (9/20)
trauma surgery	25% (5/20)
neurosurgery	20% (4/20)
vascular surgery	10% (2/20)

Data are presented as median (25th; 75th). Categorical data are presented as percentage values (frequencies).

**Table 2:** Changes of volatile organic compounds (VOCs) over the time.

Tentative VOC	(A) Baseline (0 min) (ncps)	(B) 15 min (ncps)	(C) 30 Min. (ncps)	*p* Friedman Test	*p* A vs. B	*p* A vs. C	*p* B vs. C
acetonitrile	353.0 (308.5;746.3)	324.8 (219.1;690.6)	282.2 (212.1;627.9)	<0.001	<0.001	<0.001	0.014
acetaldehyde	511.7 (341.9;1058.5)	401.6 (289.7;908.8)	386.5 (304.5;851.2)	0.002	0.002	0.001	0.074
ethanol	73.9 (58.2;142.4)	55.0 (42.8;130.4)	52.0 (40.3;111.3)	0.003	<0.001	0.003	0.18
methanethiol	665.6 (71.1;4745.8)	550.2 (49.1;4668.9)	471.1 (47.2;4152.1)	<0.001	0.004	<0.001	<0.001
acrolein	81.4 (65.1;119.0)	73.9 (54.4;101.5)	68.1 (52.1;108.3)	0.058	n.a.	n.a.	n.a.
acetone (via isotope at *m*/*z* 60)	2954.7 (1676.6;5929.1)	2595.4 (1733.3;5612.3)	2796.4 (1769.7;6038.5)	0.819	n.a.	n.a.	n.a.
acetic acid	584.0 (327.4;1063.1)	540.6 (323.4;713.0)	554.5 (312.5;913.5)	0.819	n.a.	n.a.	n.a.
propanol	641.5 (460.1;1397.1)	555.0 (330.9;981.8)	551.1 (301.4;888.4)	<0.001	<0.001	<0.001	0.655
dimethyl-sulfide	55.4 (35.1;115.0)	49.7 (37.1;113.4)	49.5 (38.3;110.5)	0.951	n.a.	n.a.	n.a.
isoprene	2312.1 (1579.8;2910.0)	2140.3 (1266.4;2736.5)	2155,2 (1486.7;2508.1)	0.142	n.a.	n.a.	n.a.
butenal	27.9 (20.4;55.6)	25.2 (19.8;43.9)	25.5 (18.8;47.0)	0.212	n.a.	n.a.	n.a.
butanal	60.8 (47.3;89.6)	61.6 (46.3;79.2)	59.4 (44.9;83.3)	0.387	n.a.	n.a.	n.a.
methylacetate	160.8 (105.7;266.8)	150.3 (130.5;204.0)	145.6 (110.3;228.3)	0.247	n.a.	n.a.	n.a.
1,2-propanediol	55.8 (21.6;147.2)	47.2 (22.4;151.2)	46.3 (23.9;154.5)	0.951	n.a.	n.a.	n.a.
benzene	21.4 (14.8;48.2)	18.7 (12.1;28.1)	15.7 (10.7;29.0)	<0.001	<0.001	<0.001	0.044
cyclohexadiene	273.1 (71.7;1325.3)	319.9 (74.7;1168.6)	285.0 (88.8;1075.9)	0.247	n.a.	n.a.	n.a.
pentanal	28.3 (18.6;44.8)	23.8 (18.6;45.6)	24.8 (18.5;46.5)	0.819	n.a.	n.a.	n.a.
ethylacetate	36.9 (23.7;77.7)	38.0 (23.9;78.1)	45.5 (22.2;73.5)	0.387	n.a.	n.a.	n.a.
1-butanethiol	25.8 (17.3;150.2)	25.6 (20.0;160.7)	23.8 (18.2;159.9)	0.004	0.655	0.008	<0.001
hexenal	76.1 (42.0;1558.9.3)	63.1 (37.4;1728.4)	53.2 (36.0;1497.3)	<0.001	<0.001	<0.001	<0.001
cyclooctadiene	63.5 (53.6;68.3)	60.0 (50.3;70.5)	57.3 (52.4;64.4)	0.142	n.a.	n.a.	n.a.
acetophenone	77.8 (64.5;104.6)	72.4 (55.7;153.4)	70.4 (55.3;142.8)	0.074	n.a.	n.a.	n.a.
benzopyran	57.0 (11.4;630.6)	52.2 (12.1;600.6)	43.6 (10.7;523.0)	0.004	0.007	0.003	0.044
limonene	27.5 (21.0;38.8)	27.1 (21.1;41.9)	26.7 (22.2;49.6)	0.116	n.a.	n.a.	n.a.

Baseline values and changes of VOCs over the time are given as median (25th; 75th percentile). ncps: counts per seconds normalized to primary ion (H_3_O^+^) values; VOC: Volatile Organic Compound; n.a.: not applicable.

**Table 3:** Changes of patients’ catecholamine plasma levels.

Catecholamine	Prior to Analgesic Treatment (nmol/L)	30 min after Analgesic Treatment (nmol/L)	*p*
epinephrine	0.31 (0.10;1.02)	0.51 (0.11;1.21)	0.492
norepinephrine	1.94 (1.52;5.77)	1.97 (1.37;3.66)	0.044
dopamine	0.19 (0.04;0.52)	0.28 (0.11;0.34)	0.557

Data are given as median (25th; 75th) percentile. Blood samples were taken prior to analgesic treatment (0 min) and 30 min after analgesic treatment.

## Supplementary Materials

The following are available online at https://www.mdpi.com/2218-1989/10/8/321/s1, Table S1: Individual pre-existing conditions and the specific indications for the performed surgeries for each patient. Table S2: Relative changes of VOCs over time. Table S3: Cardiovascular variables during analgesic treatment. Table S4: Results of correlation analysis for the variables “catecholamine levels”, “cardiovascular variables”, and “alterations of VOC levels” using Pearson’s correlation test. Figure S1: Changes in cardiovascular variables during analgesic treatment for each patient individually.
