# Supplementary material for: Changes of Exhaled Volatile Organic Compounds in Postoperative Patients Undergoing Analgesic Treatment: A Prospective Observational Study
Source: Metabolites. 2020 Aug 7;10(8):321. doi: 10.3390/metabo10080321 (PMC7463857; doi:10.3390/metabo10080321)
Supplement: Supplementary file 1 [file metabolites-10-00321-s001.zip › Figure S1.pptx]

## Slide 1
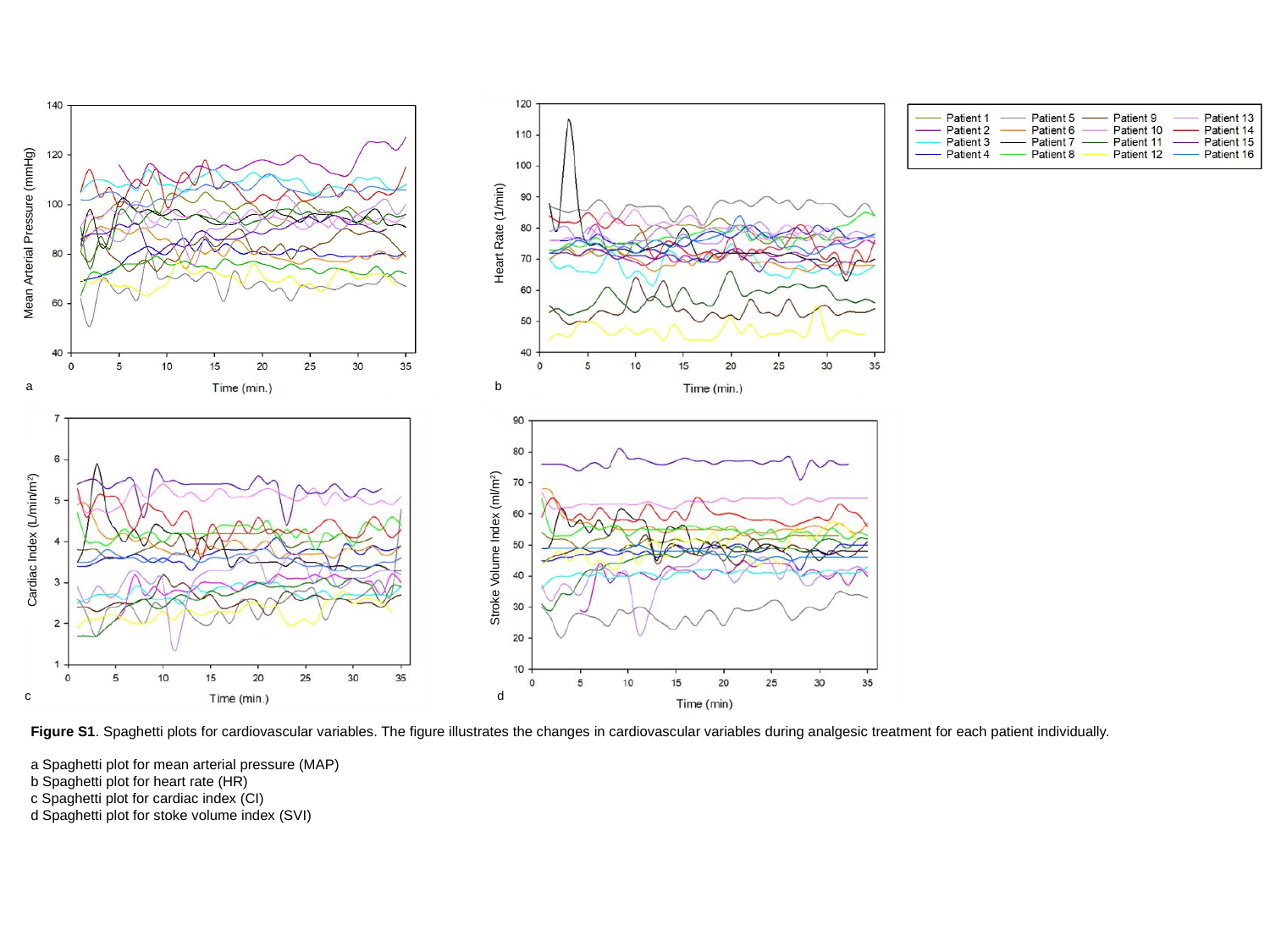

Heart Rate (1/min)
Mean Arterial Pressure (mmHg)
Cardiac Index (L/min/m2)
Stroke Volume Index (ml/m2)
a
b
c
d
Figure S1. Spaghetti plots for cardiovascular variables. The figure illustrates the changes in cardiovascular variables during analgesic treatment for each patient individually.
a Spaghetti plot for mean arterial pressure (MAP)
b Spaghetti plot for heart rate (HR)
c Spaghetti plot for cardiac index (CI)
d Spaghetti plot for stoke volume index (SVI)
