# Supplementary material for: Changes of Exhaled Volatile Organic Compounds in Postoperative Patients Undergoing Analgesic Treatment: A Prospective Observational Study
Source: Metabolites. 2020 Aug 7;10(8):321. doi: 10.3390/metabo10080321 (PMC7463857; doi:10.3390/metabo10080321)
Supplement: Supplementary file 1 [file metabolites-10-00321-s001.zip › Table S1.pdf]

Table S1 Table that demonstrates individual pre-existing conditions and the specific indications for surgery for each patient

Patient	Surgical indication	Pre-existing conditions
1	colorectal carcinoma	history of smoking
2	abdominal aortic aneurysm	obesity, arterial hypertension
3	arthrosis	obesity
4	pancreatitis	arterial hypertension, depression, history of deep vein thrombosis
5	infection after hip replacement	renal failure, arterial hypertension, factor XIII deficiency, alcohol-related liver cirrhosis, history of smoking
6	occlusion of femoropopliteal bypass	arterial hypertension, CHD, hyperlipoproteinemia, PAD
7	goiter	obesity, OSA, arterial hypertension, hyperuricemia
8	proctitis	ulcerative colitis
9	spinal disc herniation	history of smoking, alcohol abuse
10	cholecystitis	obesity
11	spinal disc herniation	obesity
12	fracture of thoracic vertebra	obesity, arterial hypertension, benign prostatic hyperplasia, depression, anxiety disorder, hypogonadism
13	necrosis of amputation stump	arterial hypertension, PAD, CHD, hyperlipidaemia, degenerative lumbar spinal syndrome, history of smoking
14	delayed wound healing (thumb)	obesity, history of smoking
15	Delayed wound healing (foot)	arterial hypertension, diabetes mellitus
16	goiter	arterial hypertension, renal failure, hyperparathyreoidism with severe hypercalcemia, steatosis hepatis, depression, benign prostate hyperplasia
17	inguinal hernia	history of smoking, polyneuropathy, venous insufficiency, atrial fibrillation, COPD, asthma, chronic heart failure, pulmonary arterial hypertension, cor pulmonale
18	spinal disc herniation	CHD, arterial hypertension, renal failure, hypothyroidism, stenosis of coeliac trunk
19	hepatic metastases	obesity, arterial hypertension, CHD, history of coronary aneurysm, PAD, hyperlipoproteinemia, COPD, OSA, axial hiatal hernia
20	tendinosis of calcaneal tendon	obesity

PAD: Peripheral Artery Disease; OSA: Obstructive Sleep Apnea; CHD: Coronary Heart Disease; COPD Chronic Obstructive Pulmonary Disease
