# Supplementary material for: Changes of Exhaled Volatile Organic Compounds in Postoperative Patients Undergoing Analgesic Treatment: A Prospective Observational Study
Source: Metabolites. 2020 Aug 7;10(8):321. doi: 10.3390/metabo10080321 (PMC7463857; doi:10.3390/metabo10080321)
Supplement: Supplementary file 1 [file metabolites-10-00321-s001.zip › Table S2.pdf]

Table S2 Table that demonstrates relative changes of VOC over time

**VOC**	(B)<br>(%) Change of normalized values after 15 min	(C)<br>(%) Change of normalized values after 30 min	P<br>Friedman	P<br>A vs B	P<br>A vs C	P<br>B vs C
acetonitrile	-18.6	-25.2	<0.001	<0.001	<0.001	0.014
acetaldehyde	-4.53	-9.93	0.002	0.001	0.002	0.118
ethanol	-12.97	-22.04	0.004	0.002	0.003	0.146
methanethiol	-8.55	-10.46	<0.001	0.004	<0.001	<0.001
acrolein	-9.41	-10.41	0.044	0.118	0.038	0.057
acetone (via isotope at m/z 60)	-1.66	-1.18	0.65	n.a.	n.a.	n.a.
acetic acid	+8.68	+14.62	0.892	n.a.	n.a.	n.a.
propanol	-23.68	-24.74	<0.001	<0.001	<0.001	0.314
dimethyl-sulfide	-1.59	-4.27	0.89	n.a.	n.a.	n.a.
isoprene	-4.57	-5.77	0.157	n.a.	n.a.	n.a.
butenal	+3.92	-1.54	0.212	n.a.	n.a.	n.a.
butanal	-3.30	-2.04	0.457	n.a.	n.a.	n.a.
methylacetate	+0.68	+3.05	0.182	n.a.	n.a.	n.a.
1,2-propanediol	-4.21	-4.01	0.987	n.a.	n.a.	n.a.
benzene	-26.02	-30.23	<0.001	<0.001	0.001	0.044
cyclohexadiene	+68.33	+35.56	0.247	n.a.	n.a.	n.a.
pentanal	-4.67	-4.31	0.856	n.a.	n.a.	n.a.
ethylacetate	+11.25	+8.27	0.387	n.a.	n.a.	n.a.
1-butanethiol	+0.73	-5.41	0.004	0.823	0.01	<0.001
hexenal	-1.11	-18.63	<0.001	<0.001	<0.001	<0.001
cyclooctadiene	-0,87	-3,71	0.164	n.a.	n.a.	n.a.
acetophenone	+7.15	-0.071	0.074	n.a.	n.a.	n.a.
benzopyran	-7.40	-11.21	0.003	0.007	0.003	0.044
limonene	+0.82	+9.64	0.158	n.a.	n.a.	n.a.

Differences of VOCs are given in percentage from baseline value with corresponding significances. VOC: Volatile Organic Compound; n.a.: not applicable
