# Supplementary material for: Changes of Exhaled Volatile Organic Compounds in Postoperative Patients Undergoing Analgesic Treatment: A Prospective Observational Study
Source: Metabolites. 2020 Aug 7;10(8):321. doi: 10.3390/metabo10080321 (PMC7463857; doi:10.3390/metabo10080321)
Supplement: Supplementary file 1 [file metabolites-10-00321-s001.zip › Table S3.pdf]

Table S3 Table that demonstrates cardiovascular variables during analgesic treatment

	(A)<br>0 min	(B)<br>15 min	(C)<br>30 min	P	P<br>(A vs B)	P<br>(A vs C)	P<br>(B vs C)
SAP (mmHg)	131 (97;138)	127 (113;147)	132 (112;140)	0.001	0.004	<0.001	0.708
DAP (mmHg)	66 (56;75)	68 (58;78)	67 (58;80)	0.655	n.a.	n.a.	n.a.
MAP (mmHg)	89 (74;101)	93 (81;104)	93 (79;104)	0.013	0.017	0.004	0.317
HR (bpm)	74 (68;79)	73 (70;79)	73 (67;78)	0.504	n.a.	n.a.	n.a.
CI (L/min/m^2)	3.6 (2.3;4.6)	3.7 (2.8;4.3)	3.4 (2.9;4.3)	0.034	0.036	0.034	0.169
SVI (ml/m^2)	48 (36;60)	50 (45;56)	50 (43;56)	0.116	n.a.	n.a.	n.a.

Data are given as median (25th;75th) percentile. SAP: Systolic Arterial Pressure; DAP: Diastolic Arterial Pressure; MAP: Mean Arterial Pressure; HR: Heart Rate; CI: Cardiac Index; SVI: Stroke Volume Index; n.a.: not applicable
