# Supplementary material for: Changes of Exhaled Volatile Organic Compounds in Postoperative Patients Undergoing Analgesic Treatment: A Prospective Observational Study
Source: Metabolites. 2020 Aug 7;10(8):321. doi: 10.3390/metabo10080321 (PMC7463857; doi:10.3390/metabo10080321)
Supplement: Supplementary file 1 [file Table S4.pdf › Table S4.pdf]

Table S4 Table that demonstrates the results of a performed a correlation analysis for the variables "catecholamine levels", "cardiovascular variables" and "alterations of VOC levels" using Pearson's correlation test.

		CO (l/min) - 0 min	CO (l/min) - 15 min	CO (l/min) - 30 min	SV (ml) - 0 min	SV (ml) - 15 min	SV (ml) - 30 min	SVV (%) - 0 min	SVV (%) - 15 min	SVV (%) - 30 min	SAP (mmHg) - 0 min	SAP (mmHg) - 15 min	SAP (mmHg) - 30 min	DAP (mmHg) - 0 min	DAP (mmHg) - 15 min	DAP (mmHg) - 30 min	MAP (mmHg) - 0 min
CO (l/min) - 0 min	Pearson Correlation	1	[illegible]	[illegible]	[illegible]	[illegible]	[illegible]	[illegible]	-,346	-,183	[illegible]	,398	,269	-,090	-,149	-,148	,325
	Sig. (2-tailed)		,000	,000	,000	,000	,002	,010	,190	,498	,029	,127	,314	,740	,582	,585	,219
	N	16	16	16	16	16	16	16	16	16	16	16	16	16	16	16	16
CO (l/min) - 15 min	Pearson Correlation	[illegible]	1	[illegible]	[illegible]	[illegible]	[illegible]	-,487	-,450	-,304	[illegible]	[illegible]	,429	,087	,005	-,020	,449
	Sig. (2-tailed)	,000		,000	,000	,000	,001	,056	,080	,252	,015	,034	,097	,747	,985	,942	,081
	N	16	16	16	16	16	16	16	16	16	16	16	16	16	16	16	16
CO (l/min) - 30 min	Pearson Correlation	[illegible]	[illegible]	1	[illegible]	[illegible]	[illegible]	-,434	-,392	-,282	[illegible]	,421	,327	-,004	-,090	-,129	,342
	Sig. (2-tailed)	,000	,000		,000	,000	,001	,093	,134	,290	,049	,104	,217	,988	,741	,635	,195
	N	16	16	16	16	16	16	16	16	16	16	16	16	16	16	16	16
SV (ml) - 0 min	Pearson Correlation	[illegible]	[illegible]	[illegible]	1	[illegible]	[illegible]	[illegible]	-,482	-,163	[illegible]	,355	,205	-,179	-,251	-,279	,251
	Sig. (2-tailed)	,000	,000	,000		,000	,000	,000	,058	,547	,045	,177	,445	,508	,349	,295	,349
	N	16	16	16	16	16	16	16	16	16	16	16	16	16	16	16	16
SV (ml) - 15 min	Pearson Correlation	[illegible]	[illegible]	[illegible]	[illegible]	1	[illegible]	[illegible]	[illegible]	-,204	[illegible]	,444	,357	,007	-,112	-,129	,357
	Sig. (2-tailed)	,000	,000	,000	,000		,000	,010	,017	,449	,040	,085	,174	,979	,680	,635	,175
	N	16	16	16	16	16	16	16	16	16	16	16	16	16	16	16	16
SV (ml) - 30 min	Pearson Correlation	[illegible]	[illegible]	[illegible]	[illegible]	[illegible]	1	[illegible]	[illegible]	-,123	,401	,317	,233	-,066	-,177	-,229	,247
	Sig. (2-tailed)	,002	,001	,001	,000	,000		,020	,044	,649	,124	,232	,384	,808	,512	,393	,356
	N	16	16	16	16	16	16	16	16	16	16	16	16	16	16	16	16
SVV (%) - 0 min	Pearson Correlation	[illegible]	-,487	-,434	[illegible]	[illegible]	[illegible]	1	[illegible]	,456	-,330	-,127	,017	,315	,397	,411	-,050
	Sig. (2-tailed)	,010	,056	,093	,000	,010	,020		,003	,076	,212	,640	,951	,235	,128	,114	,853
	N	16	16	16	16	16	16	16	16	16	16	16	16	16	16	16	16
SVV (%) - 15 min	Pearson Correlation	-,346	-,450	-,392	-,482	[illegible]	[illegible]	[illegible]	1	[illegible]	-,384	-,313	-,297	-,064	,147	,100	-,268
	Sig. (2-tailed)	,190	,080	,134	,058	,017	,044	,003		,000	,142	,239	,264	,815	,587	,714	,316
	N	16	16	16	16	16	16	16	16	16	16	16	16	16	16	16	16
SVV (%) - 30 min	Pearson Correlation	-,183	-,304	-,282	-,163	-,204	-,123	,456	[illegible]	1	-,239	-,208	-,235	-,006	,166	,077	-,145
	Sig. (2-tailed)	,498	,252	,290	,547	,449	,649	,076	,000		,372	,439	,381	,982	,538	,778	,592
	N	16	16	16	16	16	16	16	16	16	16	16	16	16	16	16	16
SAP (mmHg) - 0 min	Pearson Correlation	[illegible]	[illegible]	[illegible]	[illegible]	[illegible]	,401	-,330	-,384	-,239	1	[illegible]	[illegible]	[illegible]	[illegible]	[illegible]	[illegible]
	Sig. (2-tailed)	,029	,015	,049	,045	,040	,124	,212	,142	,372		,000	,000	,009	,036	,037	,000
	N	16	16	16	16	16	16	16	16	16	16	16	16	16	16	16	16
SAP (mmHg) - 15 min	Pearson Correlation	,398	[illegible]	,421	,355	,444	,317	-,127	-,313	-,208	[illegible]	1	[illegible]	[illegible]	[illegible]	[illegible]	[illegible]
	Sig. (2-tailed)	,127	,034	,104	,177	,085	,232	,640	,239	,439	,000		,000	,001	,005	,006	,000
	N	16	16	16	16	16	16	16	16	16	16	16	16	16	16	16	16
SAP (mmHg) - 30 min	Pearson Correlation	,269	,429	,327	,205	,357	,233	,017	-,297	-,235	[illegible]	[illegible]	1	[illegible]	[illegible]	[illegible]	[illegible]
	Sig. (2-tailed)	,314	,097	,217	,445	,174	,384	,951	,264	,381	,000	,000		,000	,002	,001	,000
	N	16	16	16	16	16	16	16	16	16	16	16	16	16	16	16	16
DAP (mmHg) - 0 min	Pearson Correlation	-,090	,087	-,004	-,179	,007	-,066	,315	-,064	-,006	[illegible]	[illegible]	[illegible]	1	[illegible]	[illegible]	[illegible]
	Sig. (2-tailed)	,740	,747	,988	,508	,979	,808	,235	,815	,982	,009	,001	,000		,000	,000	,000
	N	16	16	16	16	16	16	16	16	16	16	16	16	16	16	16	16
DAP (mmHg) - 15 min	Pearson Correlation	-,149	,005	-,090	-,251	-,112	-,177	,397	,147	,166	[illegible]	[illegible]	[illegible]	[illegible]	1	[illegible]	[illegible]
	Sig. (2-tailed)	,582	,985	,741	,349	,680	,512	,128	,587	,538	,036	,005	,002	,000		,000	,000
	N	16	16	16	16	16	16	16	16	16	16	16	16	16	16	16	16

[illegible]

Results of a performed correlation analysis for the variables "catecholamine levels", "cardiovascular variables" and "VOC levels". SAP: Systolic Arterial Pressure; DAP: Diastolic Arterial Pressure; MAP: Mean Arterial Pressure; HR: Heart Rate; SV: Stroke Volume; SVV: Stroke Volume Variance; * Correlation is significant at the 0.05 level (2-tailed); ** Correlation is significant at the 0.01 level (2-tailed)
